# O15 Tumour-induced osteomalacia: a diagnostic challenge

**DOI:** 10.1093/rap/rkab067.014

**Published:** 2021-10-19

**Authors:** Salema Khalid, Kassim Javaid, Neil Buchanan

**Affiliations:** 1 Royal Hampshire County Hospital, Winchester, United Kingdom; 2 Oxford University Hospital, Oxford, United Kingdom

## Abstract

**Case report - Introduction:**

Tumor-induced osteomalacia is a rare acquired metabolic bone disorder characterised by isolated renal phosphate wasting due to abnormal tumour production of fibroblast growth factor 23 (FGF23). Bloods show hypophosphatemia. However, given the lack of awareness of this disease, and the non-specific nature of the presenting symptoms, diagnosis is often delayed for years or even missed.

With this case report, we want to shed light on this rare bone disorder and highlight the importance of not ignoring a low phosphate level.

**Case report - Case description:**

We report the case of a 63-year-old gentleman who presented to us with a history of progressive bone pain, marked muscle weakness and fatigue. He had a persistently raised parathyroid hormone and very high alkaline phosphatase. Calcium was low normal, but vitamin D was normal. Phosphate was noted to be low and had been so since 6 years prior to being referred. X-ray pelvis showed sclerotic-looking iliac bones, but an isotope bone scan showed no evidence of metastatic disease.

We referred him to our endocrinology colleagues to investigate for possible primary hyperparathyroidism.  However, ultrasound scan of neck showed no abnormal parathyroid gland. PET and CT scan of the chest, abdomen and pelvis did not show any significant pathology.

MIBI scan of parathyroid glands showed diffuse thyroid uptake. The SPECT-CT images showed a well-defined focal area of increased activity, posterior to the mid/lower pole of the right thyroid lobe, possibly indicative of hyperfunctioning parathyroid tissue. The radiologist questioned the possibility of renal osteodystrophy and resultant secondary hyperparathyroidism. However, there was no evidence for renal dysfunction with a creatinine of 80 umol/L.

We referred this gentleman to the Oxfordshire Osteoporosis and Metabolic Bone Service. Bloods showed a raised P1NP. This was felt to be consistent with tumour-induced osteomalacia. Oversecretion of FGF23 was confirmed and DOTATATE scan revealed two areas of uptake: one in the right hip and one in the skull.

Meanwhile, the patient had been started on Sandoz Phosphate and alfacalcidol, and his bone aches improved on this combination therapy.

He had radio-ablation of the hip lesion this year and has now stopped all his medication. His fatigue and muscle strength has improved dramatically. The skull lesion is being monitored for now with yearly imaging and follow-up in the metabolic bone clinic.

**Case report - Discussion:**

Tumour-induced or oncogenic osteomalacia is extremely rare. Symptoms include chronic muscle and bone pain, weakness, and fatigue and can result in fragility fractures due to osteomalacia. Lack of awareness of these clinical manifestations and relevance of low phosphate levels often leads to a diagnostic delay or misdiagnosis. As seen in our case, it took multiple investigations and almost 2 years before this gentleman had a confirmation of his diagnosis.

The pathogenesis of tumour-induced osteomalacia involves tumour expression of fibroblast growth factor 23. This hormone inhibits reabsorption of phosphate in the proximal renal tubule and down-regulates renal conversion of 25-hydroxyvitamin D to its active form, 1,25-dihydroxyvitamin D. Replacing phosphate on its own paradoxically increases PTH levels. The metabolic abnormalities may be partially or completely corrected with phosphate supplementation and calcitriol. In our patient, his PTH levels remained elevated, but alkaline phosphatase improved, as did calcium and phosphate levels.

Unlike many other causes of osteomalacia, tumour-induced osteomalacia is curable by resection of the offending tumour. However, finding the responsible FGF23-secreting tumour can be incredibly challenging. There is ongoing research into medical treatment for this condition, especially if the tumour is not found or is unresectable, or the patient is not a surgical candidate.

**Case report - Key learning points:**

Low phosphate levels can be easily overseen, with significant consequences. This case emphasises the need to recognise hypophosphatemia and to look for its cause.  Conversely, it also illustrates the need to investigate all patients with persistent musculoskeletal symptoms for hypophosphatemia.

Our case underscores the importance of being aware of the rare condition of tumour-induced osteomalacia. This will enable early recognition and treatment of the metabolic abnormality, before the development of any deleterious effects of osteomalacia on the skeleton.

